# Anatomical barriers in the right atrium to the coronary sinus cannulation

**DOI:** 10.7717/peerj.1548

**Published:** 2016-01-07

**Authors:** Wiesława Klimek-Piotrowska, Mateusz K. Hołda, Mateusz Koziej, Marcin Strona

**Affiliations:** 1 Department of Anatomy, Jagiellonian University, Cracow, Poland; 2 Department of Forensic Medicine, Jagiellonian University, Cracow, Poland

**Keywords:** Thebesian valve, Chiari’s network, Tendon of Todaro, Eustachian valve, Heart anatomy

## Abstract

**Background.** The coronary venous system is an increasingly frequent target of minimally invasive cardiac procedures. The purpose of this paper is to assess the anatomical barriers in the right atrium to coronary sinus cannulation.

**Methods.** We examined the anatomy of the right atrium, coronary sinus ostium, inferior and superior vena cava ostia in 110 randomly selected autopsied human hearts of both sexes (27% females; mean age 49.2 ± 17.5 years).

**Results.** The Eustachian valve was present in 79 cases (71.8%) with mean height =4.9 ± 2.6 mm. The valve was perforated in 11 cases (13.9%). It is typically too small to hinder the coronary sinus catheterization, but in some cases (about 2%) a significantly protruding valve may be an obstacle. Chiari’s network (4.6%) is not a barrier to catheter entry into the right atrium but may significantly impede further catheter manipulations inside the heart venous system. A typical Thebesian valve leaves enough space for the passage of the standard catheter to the coronary sinus.

**Discussion.** Detailed anatomy of various anatomical structures within the right atrium that could play a potential role in coronary sinus cannulation is discussed.

## Introduction

The coronary venous system is an increasingly frequent target of minimally invasive cardiac procedures. Cardiac resynchronization therapy, catheter ablation of cardiac arrhythmias, defibrillation, perfusion therapy, percutaneous valvular interventions, targeted drug delivery, and retrograde cardioplegia administration (Spencer, Anderson & Iaizzo, 2013) are commonly used therapeutic methods that utilize coronary sinus (CS) cannulation. Access to coronary venous system is possible through the coronary sinus ostium (CSO) into the right atrium. Minimally invasive percutaneous entry into the right atrium is allowed by the inferior vena cava (IVC) and superior vena cava (SVC).

During right atrium catheterization, the operator may encounter a number of anatomical barriers that are mainly embryological remnants of the right venous valve (Sadler & Langman, 2011), including: the Eustachian valve (valve of the IVC ostium) and ridge, Chiari’s network, Thebesian valve (valve of the CSO), muscle bridges within right atrium, overdeveloped tendon of Todaro, and other networks and structures in the right atrium and ostia of main veins. The purpose of this paper is to assess the anatomical barriers to CS cannulation through main veins and right atrium.

## Materials & Methods

We examined the anatomy of the right atrium, CSO, IVC and SVC ostia in 110 randomly selected autopsied adult human (Caucasian) hearts of both sexes (27% females) with mean age =49.2 ± 17.5 years old. The study was approved by the Bioethical Committee of the Jagiellonian University Medical College, Cracow (KBET/51/B/2013). We collected hearts only from deceased persons who did not express objection, when alive, and only if the family did not also express objection. Our Bioethical Committee waived the need for consent. These samples were not procured from a tissue bank or donation center.

Specimens were collected for this study during routine forensic medical autopsies. Randomization was performed by a lottery of autopsy numbers. The hearts were removed together with the proximal portions of the great vessels: the ascending aorta, pulmonary trunk, superior vena cava, inferior vena cava, and all the pulmonary veins. Exclusion criteria included: cardiac death, anatomical abnormalities, obvious macroscopic pathology of the heart or vascular system found during the autopsy, heart trauma, and macroscopic signs of decomposition of cadavers. Hearts were weighted before fixation using a 0.5 g precision electronic laboratory scale (BSA-L Laboratory). After dissection, all hearts were fixed in 10% paraformaldehyde for a maximum of two months until the time of measurement.

All heart specimens were opened by an incision extending from the orifice of the superior vena cava to the orifice of the inferior vena cava with the exception that the Eustachian valve was not sectioned. The CS was opened longitudinally along its free wall to allow easy measurement of the diameter of the CSO without damaging present Thebesian valve. All descriptions and measurements were undertaken with the heart held in anatomical position. The presentation and morphology of the Eustachian valve, Eustachian ridge, Chiari’s network, Thebesian valve, muscle bridges within right atrium, tendon of Todaro, and other networks and structures in right atrium and ostia of main veins was macroscopically assessed.

All measurements were conducted using a 0.03 mm precision electronic caliper YATO (YT–7201). Measurements were performed twice to reduce the chance of error. If the measurements of one parameter differed by more than 10% between two researches then they were not included in the database and the sample was measured again. The mean of the two measurements was calculated, rounding to the one decimal place. The following measurements were made:

– the diameter of the IVC and SVC ostia were measured in two lengths: mediolateral and anteroposterior diameter;
– the height of the Eustachian valve was measured as the length between the free edge of the valve and its attachment site to the right atrium;
– the transverse diameter of the CSO;
– the height of Thebesian valve (between the free edge of the valve and its attachment site to the right atrium as the shortest dimension passing through the middle of the free edge parallel to the transverse diameter of the CSO).

Mean horizontal cross-sectional area of the IVC ostium, as well as mean percent coverage of this surface by the Eustachian valve was computed. The mean percentage coverage of the CSO by the Thebesian valve was calculated as the Thebesian valve height divided by the CSO diameter. StatSoft Statistica 10.0 for Windows was used for all statistical analyses. *P*-values less than 0.05 were considered to be statistically significant.

Manipulations with standard blunt, smooth electrocardiological catheters (size 7Fr and 8Fr) were performed in every heart before and after heart dissections. The purpose of manipulations was the introduction of a catheter into the coronary sinus through the present barriers and to compare the dimensions of catheter and structures within right atrium.

**Figure 1:**
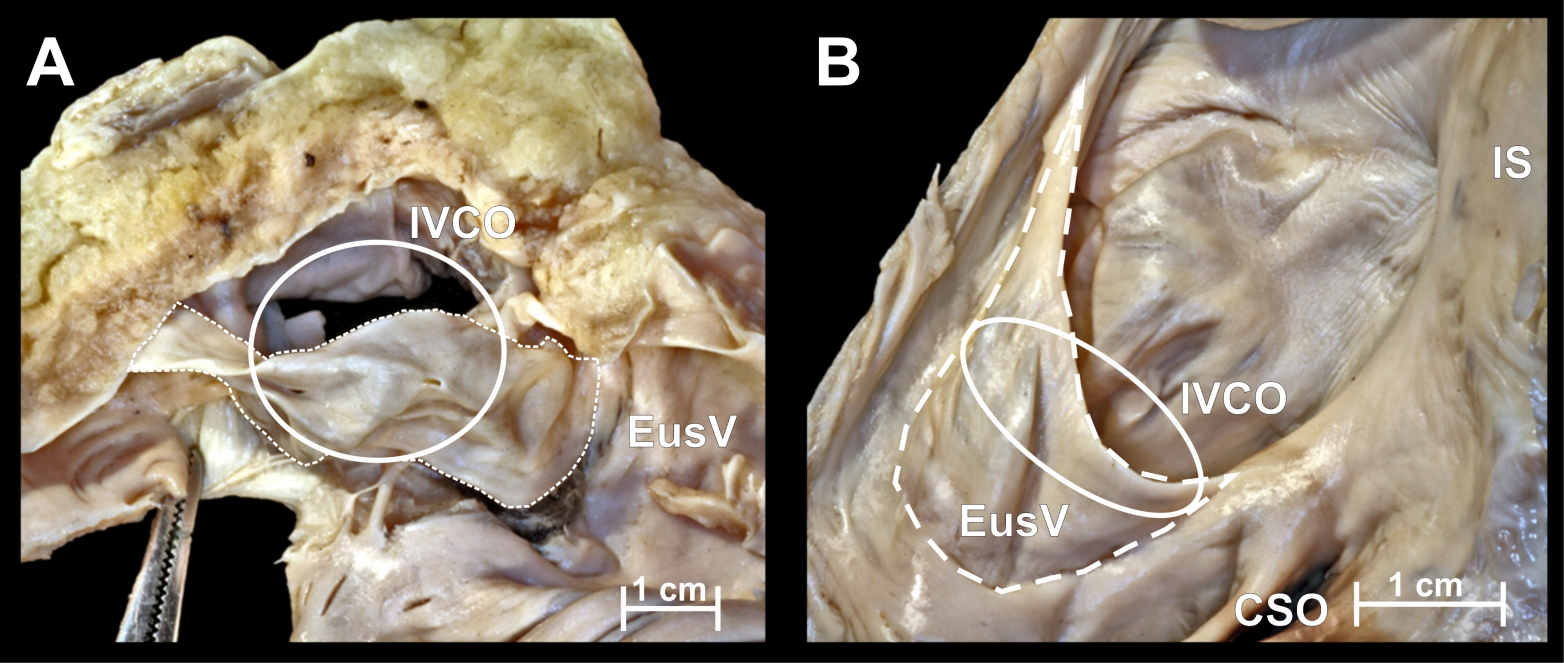
Photographs of cadaveric heart specimen. View of the inferior vena cava ostium (IVCO) with present Eustachian valve (EusV).

## Results

### Inferior vena cava ostium and Eustachian valve

Mean values of mediolateral and anteroposterior IVC diameters were 24.1 ± 5.7 mm and 24.2 ± 5.5 mm respectively. The Eustachian valve, (Fig. 1) was present in 79 cases (71.8%) with mean height 4.9 ± 2.6 mm. The valve was perforated in 11 cases (13.9%). Mean horizontal cross-sectional area of the IVC ostium was 4.8 ± 2.0 cm^2^ and mean percent coverage of this surface by the Eustachian valve was 22.9 ± 14.6%. Surface area of the IVC was positively correlated with weight of the heart (*r* = 0.27; *p* < 0.01). The diameter of the IVC ostium is relatively large compared to the size of the catheter usually introduced into the right atrium. The Eustachian valve is typically too small to hinder the catheterization, but in some cases a significantly protruding valve (the largest Eustachian valves covered almost the whole IVC ostium—two cases = 1.8%) may be a significant obstacle. The Eustachian valve directs the catheter superiorly and anteriorly (to the interatrial septum). This can lead to difficulty in accessing the CSO (located anteriorly and slight inferiorly to the valve).

### Chiari’s network

**Figure 2:**
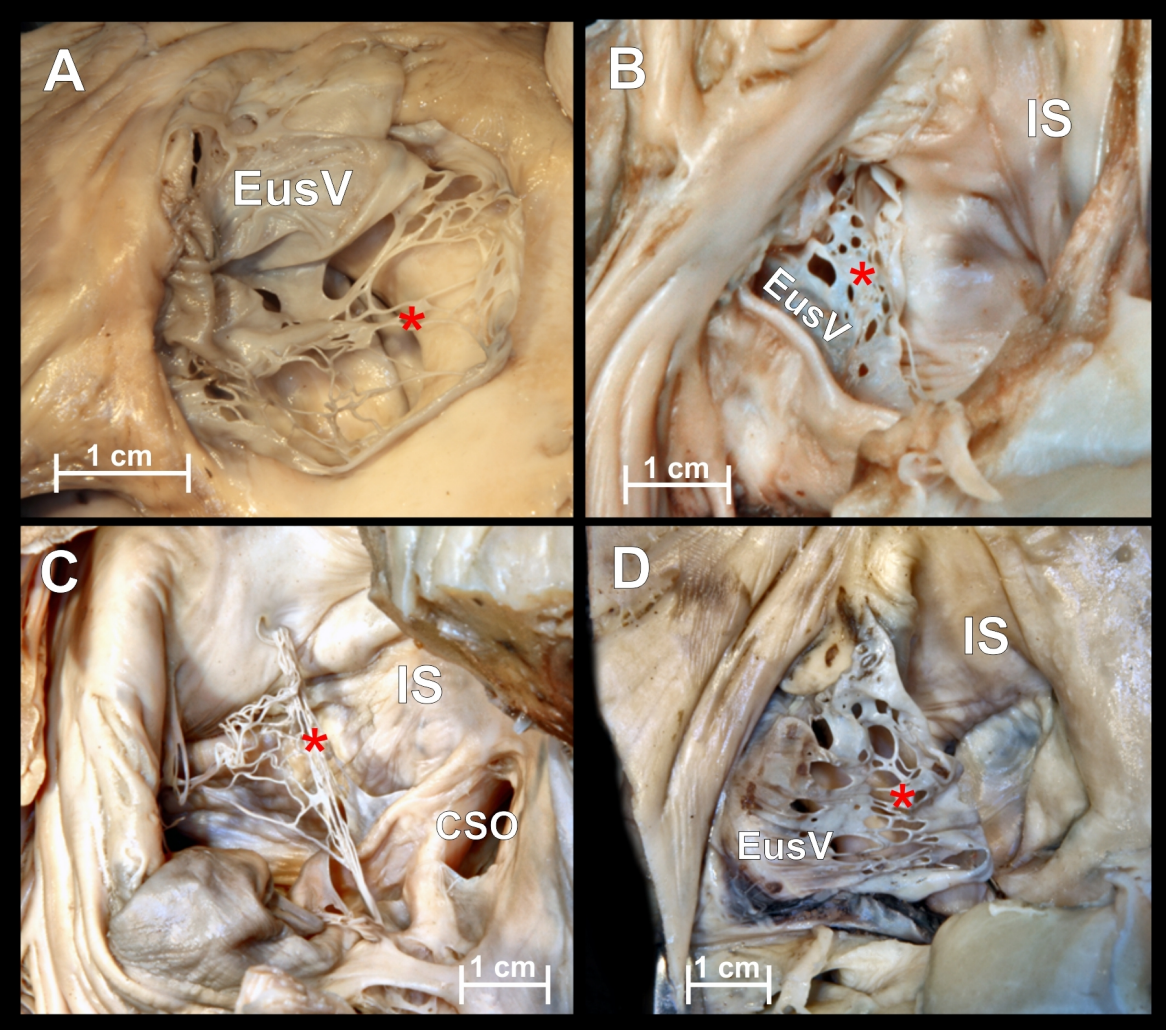
Chiari’s network. Photographs of cadaveric heart specimen. View of the inferior vena cava ostium with present Chiari’s network.

Chiari’s network is an embryologic remnant resulting from incomplete resorption of the right venous sinus valve (Fig. 2). In our study it was present in 5 hearts (4.6%) and was always accompanied by a Eustachian valve. Due to its perforated construction, the presence of a Chiari’s network should not be a barrier to standard electrophysiological catheter entry into the right atrium but may significantly impede its further manipulation. Small fenestrations of Chiari’s network (from 1 to 13 mm in diameter) form compartments through which wide catheters and catheters with additional equipment cannot be passed. In 2 cases (1.8%) strands in the IVC ostium were observed.

### Superior vena cava ostium

Mean values of mediolateral and anteroposterior SVC diameters were 20.1 ± 3.2 mm and 19.2 ± 3.1 mm respectively. The SVC ostium was always free of any anatomical barriers (muscular bridges, endocardial strands and networks), making it potentially the best place for conducting the catheter to the right atrium. Catheterization with access via the IVC provides considerably more obstacles.

### Eustachian ridge, tendon of Todaro and muscular bridges

The Eustachian ridge was observed in all cases. Since the Eustachian ridge is located directly above and slight posteriorly to the CS ostium, a more prominent ridge could be an obstacle for both IVC and SVC access. The tendon of Todaro is a collagenous band within the subendocardium and forms a continuation of the Eustachian valve/ridge. In our study it was macroscopically observed only in one case (prominent tendon). The tendon of Todaro, even overdeveloped, seems to have no effect on the CS catheterization. We did not observe muscle bridges within the right atrium, and other networks (except Chiari’s network) and structures in the right atrium and ostia of main veins.

**Figure 3:**
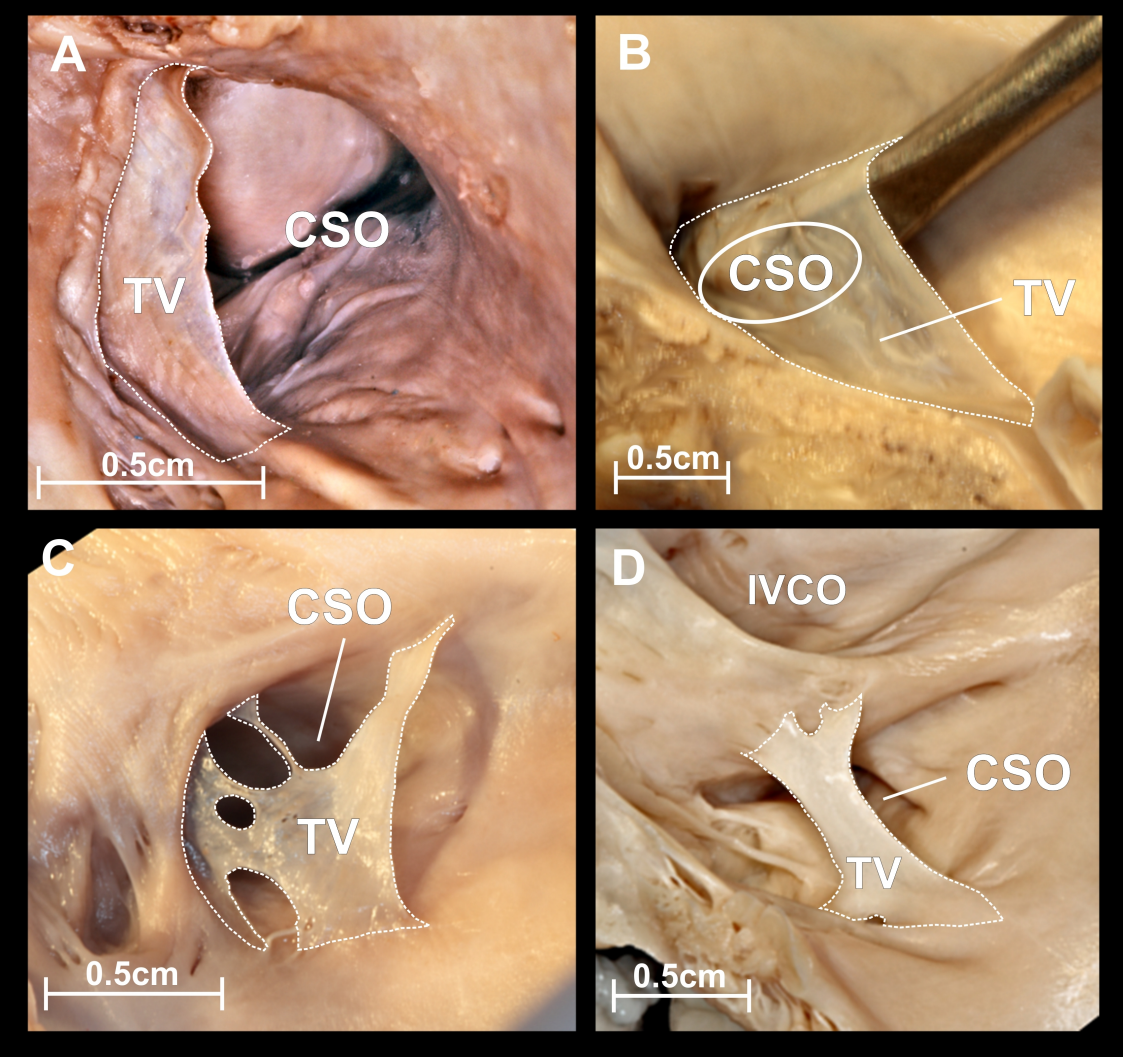
Thebesian valve. Photographs of cadaveric heart specimens. View of the coronary sinus ostium (CSO) with present Thebesian valve. (A) Thebesian valve in the form of a fold of endocardium; (B) very large fold type, covering the entire CSO, going well beyond its contour; (C) mesh or fenestrated type; (D) cord type.

### Coronary sinus ostium and Thebesian valve

The CSO was elliptic with the mean diameter (transverse diameter/long axis) amounted 9.6 ± 2.6 mm. The Thebesian valve, which is also an embryologic remnant of the right venous sinus valve was present in 97 (88.2%) cases with mean height equal to 4.8 ± 2.9 mm. In the largest number of cases (60.0%) the Thebesian valve appeared in the form of a fold of endocardium attached to CSO contour (Fig. 3A). The mean percentage coverage of the CSO by the Thebesian valve was 48.3 ± 36.6%. In 6 (5.4%) cases the Thebesian valve covered the whole CS orifice, going well beyond the CSO contour (Fig. 3B). The Thebesian valve shape was mesh or fenestrated (Fig. 3C) in 20 (18.2%) heart specimens. The cord type (Fig. 3D) was observed in 10.9% of cases, and was mostly localized in the midline of the CSO.

The overwhelming majority of the Thebesian valves took their origin from the right margin of the CSO, then extended along the caudal and cranial edge. A typical Thebesian valve leaves enough space for the passage of the standard catheter to the CS. Valves which cover the entire CSO are challenging and could make CSO access impossible. Mesh or fenestrated Thebesian valves should not be a barrier to CS cannulation but may significantly impede further catheter manipulations inside the venous system of the heart. Very often, as in the case of Chiari’s network, a catheter could be immobilized in one compartment of the network. Cord valves are not a barrier for standard catheters.

## Discussion

Catheter engagement of the CS ostium requires the alignment of the catheter tip with a small target with a diameter less than 1 cm. The occurrence of any unwanted structures, anatomical variations or pathological changes exposes the patient to a number of complications. They are associated with longer duration of procedural time, longer time of exposure to X-rays, perforations and dissections within the heart atria and heart venous system, and different access-site complications.

The Eustachian valve lies at the junction of the inferior vena cava and right atrium. In fetal life, relatively large Eustachian valve helps direct the flow of oxygenated blood through the right atrium and foramen ovale into the left atrium and away from the right ventricle. In adult life, the Eustachian valve is a semilunar fold of endocardium arising from the anterior rim of the IVC orifice. Such location of the valve directs the catheter insertion via the IVC superiorly and anteriorly. General cardiac complications associated with a large Eustachian valve include: obstruction of the inferior vena cava, thrombosis, pulmonary and paradoxical embolization (Schuchlenz et al., 2004). The IVC and CS ostia may be completely separated by a ridge of tissue—a Eustachian valve, which may make CSO cannulation via the IVC very difficult. In such cases, an elastic resistance at the level of the lower aspect of the right atrium could be perceptible. Moreover, a prominent Eustachian valve can also interfere with the occluder device during transcatheter patent foramen ovale closure (Roelandt & Budts, 2008; Skowasch et al., 2004). An Eustachian valve is present in about 72% of cases, but it could be visualized in only 0.20% adults undergoing routine echocardiographic examinations and can be mistaken for an atrial septal defect (Sarupria et al., 2014). Intracardiac and transesophageal echocardiography proved to be a more valuable tool during venous cannulation because they reveal the presence of a prominent, clinically significant Eustachian valve (Bencsik, Pap & Sághy, 2009; Schmid et al., 2010). To avoid an obstacle valve we can use SVC access. If superior access is not possible the use of radiofrequency energy to traverse an occlusive valve, as in the cases of transseptal access across the fossa ovalis or needle puncture of the valve, may be used as alternative methods.

Chiari’s network is a mobile, net-like structures and diagnosed by the presence of fibers originating from the Eustachian or Thebesian valve with attachments to the interatrial septum or crista terminalis, with a prevalence of 2.0% in transesophageal echocardiography (Schneider et al., 1995). In an autopsy study of 213 hearts the Chiari’s network is described with a frequency of 13.6% (Bhatnagar et al., 2006), compared to 4.6% in this study, which suggests that it may be under-diagnosed in transesophageal echocardiography studies. It has been reported that the Chiari’s network can hinder catheterization of the right heart and coronary sinus. This condition may require withdrawal and rotation of the catheter, which significantly prolongs the procedure time. Chiari’s network could be easily imaged using transthoracic echocardiography (as a highly mobile, highly reflectant echo structure). When Chiari’s network causes an obstruction, transesophageal echocardiography offers a valuable resource in assisting the operator (Teo, Ittleman & Hamlin, 2010). Moreover Chiari’s network could be seen in computed tomography and magnetic resonance techniques (Loukas et al., 2010).

The presence and shape of the Thebesian valve is the subject of many previous studies (Anh et al., 2008; Gami et al., 2010; Ghosh, Raheja & Tuli, 2013; Mak et al., 2009; Noheria et al., 2013). Clinical data point to about 3.0% risk of failures in the CSO cannulation due to inability to locate the CSO, probably because of the prominent Thebesian valve (Azizi et al., 2006; Gras et al., 1998). Complications accompanying CSO catheterization such as coronary venous dissection and perforation of the CS or cardiac veins occur respectively in 2.88% and 1.2% of cases (Azizi et al., 2006). Lesions of the CS are very difficult or even impossible to repair and can be fatal (Noble et al., 2011). Such perforations and dissections may be a direct consequence of the use of excessive force when guiding the catheter through the CSO in which a prominent Thebesian valve is present. In our previous report, we concluded that only Thebesian valves that cover >100% of coronary sinus ostium (going well beyond the CSO contour) can be established as obstructive vales and can make CS cannulation impossible (2.6% of all cases, which corresponds to clinical data) (Hołda et al., 2015). In other cases, the Thebesian valve should only be considered as hindering the CS cannulation. Access to the heart venous system via the SVC and the left superior margin of the CSO (inserting the catheter from anterior to posterior and from left to right side with a rotational movement) can significantly reduce the risk of perforation associated with overcoming the prominent Thebesian valve. Contrast injection may allow visualization of the valvular problem and help to solve it. Moreover, the presence of an obstructive Thebesian valve does not rule out the possibility of successful CS cannulation: for example, the use of radiofrequency energy to traverse an occlusive Thebesian valve, may be used as an alternative method (Parikh et al., 2011).

Cardiac venous system catheterization via the SVC requires a radial, internal jugular, or subclavian approach to the right atrium. The advantage of transradial heart catheterization is reduced access-site complications, including reduced bleeding complications. On the other hand, femoral arterial cannulation, which is required in IVC access, carries a significant risk of access-site bleeding complications. Using radial region approaches, these bleeding risks appear substantially diminished and the vascular access can be removed immediately at the conclusion of its use. Moreover, even patients with therapeutic levels of anticoagulation can undergo radial catheterization (Gilchrist et al., 2002). For these reasons, procedures that could potentially be done without femoral artery access should be done with different approaches. We should not forget that right subclavian and left internal jugular approaches introduce additional curves that could complicate catheter alignment and advancement (Miller, 2011).

The main limitation of this study is that all the measurements and observations were made on autopsied heart specimens that have been fixed in formalin, which could cause some slight changes to the size and shape of the heart and relevant structures. Studies performed on post-mortem material may not directly correlate to the physiology of tissues *in vivo*. Therefore, we cannot say anything about behavior and dimension changes of the right atrium, main veins and the area of the CSO within the cardiac cycle. Despite these limitations, we believe that they do not impede our morphological analysis. It must be emphasized, that the choice of the appropriate access to the coronary sinus should be carried out based on a comprehensive assessment of the patient’s anatomy and clinical condition, available equipment and operator’s experience.

## Supplemental Information

10.7717/peerj.1548/supp-1 Supplemental Information 1 Results of the measurements

## Additional Information and Declarations

### Data Availability

The following information was supplied regarding data availability: The research in this article did not generate any raw data.
