# Supplementary figures and images for: Anatomical barriers in the right atrium to the coronary sinus cannulation
Source: PeerJ. 2016 Jan 7;3:e1548. doi: 10.7717/peerj.1548 (PMC4731008; doi:10.7717/peerj.1548)

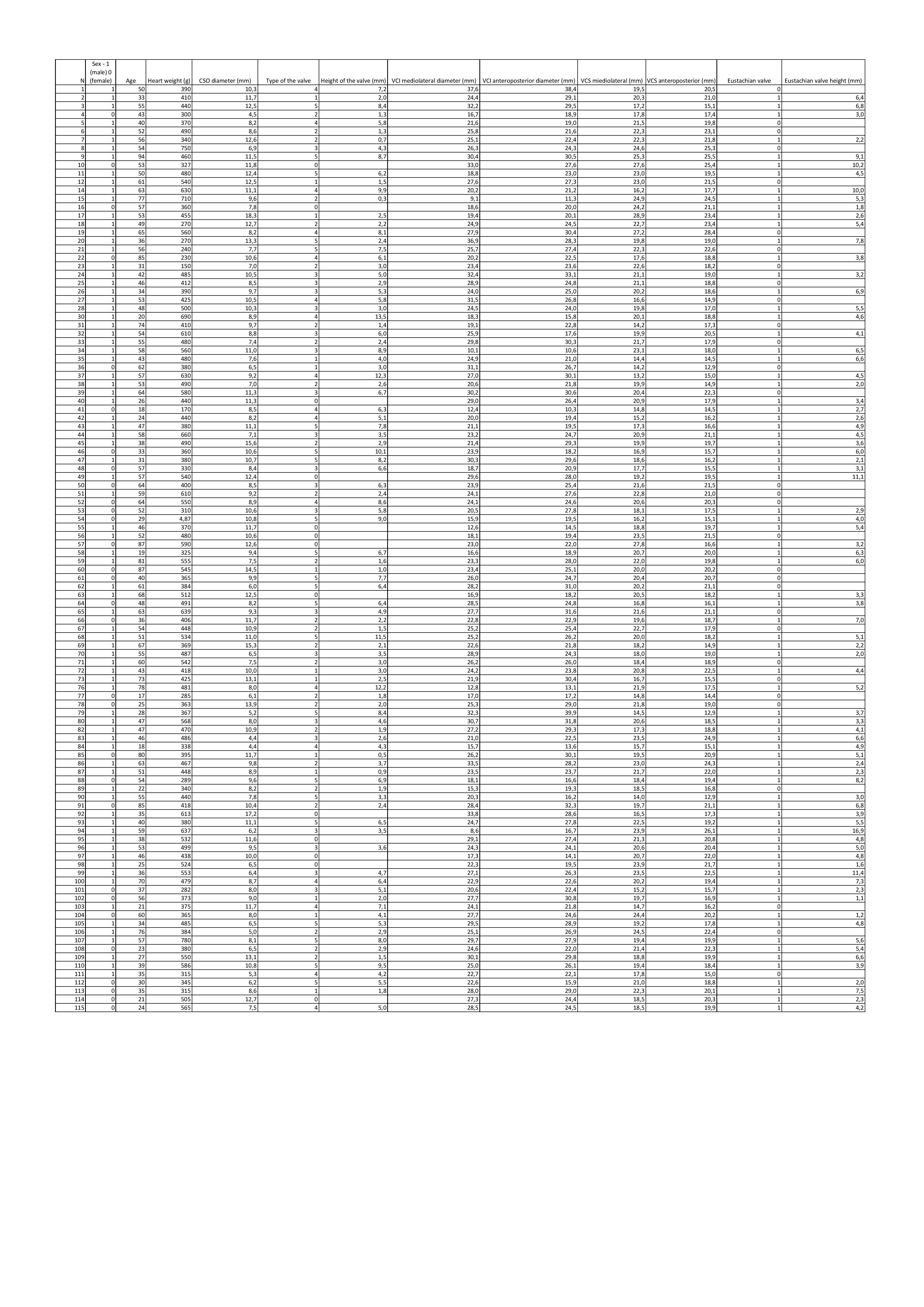

Supplement: Supplemental Information 1 [file peerj-03-1548-s001.jpg]
